# Factors predicting age at menopause among Iranian women in the Bandare-Kong cohort study (a cross-sectional survey of PERSIAN cohort study)

**DOI:** 10.1186/s40695-023-00088-z

**Published:** 2023-08-29

**Authors:** Maryam Azizi Kutenaee, Sareh Dashti, Shideh Rafati, Mehrsa Moannaei, Mojtaba Masoudi, Abdolazim Nejatizadeh, Mehdi Shahmoradi, Nasibeh Roozbeh

**Affiliations:** 1 Fertility and Infertility Research Center, Hormozgan University of Medical Sciences, Bandar Abbas, Iran; 2 Department of Public Health, Faculty of Paramedicine, Mashhad Medical Sciences, Islamic Azad University, Mashhad, Iran; 3 Department of Midwifery, Faculty of Nursing and Midwifery, Mashhad Medical Sciences, Islamic Azad University, Mashhad, Iran; 4 Social Determinants in Health Promotion Research Center, Hormozgan University of Medical Sciences, Bandar Abbas, Iran; 5 Department of Gynecology and Obstetrics, School of Medicine, Fertility and Infertility Research Center, Hormozgan University of Medical Sciences, Bandar Abbas, Iran; 6 Fatemiyeh Shiraz Institute of Higher Education, Shiraz, Iran; 7 Molecular Medicine Research Center, Hormozgan Health Institute, Hormozgan University of Medical Sciences, Bandar Abbas, Iran; 8 Endocrinology and Metabolism Research Center, Hormozgan University of Medical Sciences, Bandar Abbas, Iran; 9 Mother and Child Welfare Research Center, Hormozgan University of Medical Sciences, Bandar Abbas, Iran

**Keywords:** Menopause, Menopausal age, Women

## Abstract

**Background:**

Menopause is a natural period in women’s life and can be affected by several factors. The aim of this study was to identify the associated factors for age of natural menopause and among women with early and premature menopause based on a cohort study in Iran.

**Methods:**

This population-based study was conducted on 894 post menopause women between 35 and 70 years old who participated in the Bandare-Kong Non-Communicable Diseases (BKNCD) Cohort Study, a part of Prospective Epidemiological Research Studies in Iran (PERSIAN) from March 2016 to February 2019. All women completed a standard self-reported questionnaire. Data were analyzed using chi-square test, independent t test, and ANOVA as well as a multivariable linear regression model.

**Results:**

The mean age at natural menopause was 48.31 ± 6.34 years. After adjusting other variables, gravida, history of cardiac disease, socioeconomic status and residence status were predictive of age at menopause (P < 0.001). Among the premature menopause group, the mean age at menopause was significantly higher among women with diabetes compared to women without diabetes group (35.68 ± 2.92 vs. 33.82 ± 3.06; P = 0.043), while the mean age at menopause was significantly lower in women with infertility compared to women without infertility (29.13 ± 5.22 vs. 34.84 ± 2.826; P = 0.048).

**Conclusions:**

This study suggests that the predictors of menopausal age differed in women with premature menopause compared to overall menopause age. Prospective studies are needed to evaluation the effects of these factors on menopausal age.

**Supplementary Information:**

The online version contains supplementary material available at 10.1186/s40695-023-00088-z.

## Background

Menopause is defined as loss of menstruation and is the transition period from reproductive to non-reproductive state due to ovarian failure in women. Estrogen levels decrease and eventually lead to the menstrual cessation. This natural biological process usually occurs between 40 and 55 years old with the median of 51 years old [1, 2]. Previous studies have reported that menopause can influence the function of immune, cardiovascular, skeletal, endocrine, and genitourinary systems [3–5]. Menopause symptoms can include both short-term symptoms, including hot flashes and night sweats (vasomotor symptoms, VMS), and chronic long-term conditions, including cardiovascular disease (CVD), osteoporosis and menopausal symptoms [6]. Based on the World Health Organization (WHO) report, approximately 1.2 billion women are menopaused in the world and 47 million women become menopaused every year [3]. Common menopause symptoms include joint pain, hot flushes, night sweats, headache, urinary tract and vaginal problems, osteoporosis, irritability or increased anxiety, dyspareunia, cardiovascular diseases and amenorrhea and hypo estrogenism [4, 5].

Menopausal age varies in different geographical regions due to environmental and socioeconomic variables, lifestyle and quality of life. In the United States, the average age of menopause is 51 years [7]. However, the median age of natural menopause is reported to range between 48 and 54 years in majority of European women [7]. The natural menopausal age among Asian women is reported to range between 49 and 51 years old. Furthermore, oral contraceptives, menarche age, calcium and vitamin D intake, genetic factors, diet, alcohol consumption, and obesity can affect menopausal age. Physical, behavioral characteristics and sociodemographic characteristics should be taken into account in the determination of the age at natural menopause. The mean menopausal age of Iranian women has been reported to range between 46.9 and 49.6 years in different parts of Iran [8–11].

Premature menopause is an important menopause associated issue. Premature menopause is related to ovarian estrogen deficiency due to decrease in hormonal secretion that occur earlier than the established age of menopause [4]. The etiology of premature menopause is still unclear. Environmental and behavioral factors, obesity, ethnicity, smoking at younger age, cultural context, biological and social factors may play a role in the onset of early or premature menopause. In addition, exposure to endocrine disruptors is constantly increasing, which may reduce ovarian reserve and accelerate normal menopause [12]. A systematic review in 2016 reported that early or premature menopause were associated with an increased risk of ischemic stroke [13]. Given the importance of early and premature menopause, evaluation of factors related to these conditions is necessary. There is scarcity of data about predictors of the age of menopause onset in south of Iran. In the present large-scale population-based study we sought to determine the age of menopause and explore the predictors of the age of natural, early, and premature menopause in women who lived in South of Iran.

## Materials and methods patients and design

### Study design

**Fig. 1:**
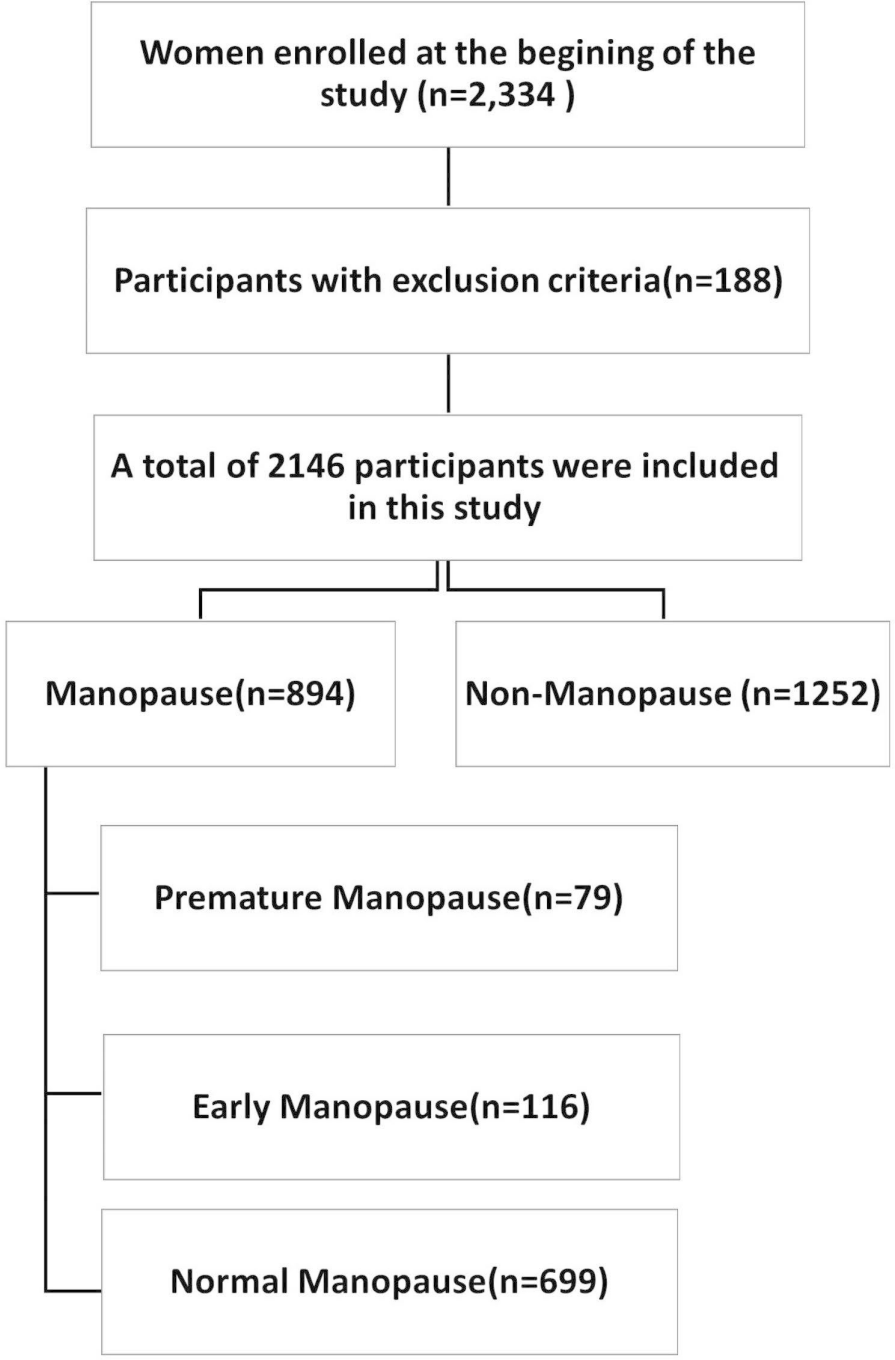
Flowchart of the sampling procedure

This cross-sectional population-based study was conducted on the data collected in the Bandare-Kong Noncommunicable Diseases (BKNCD) cohort. BKNCD is a prospective cohort study that was conducted from March 2016 to February 2019 on 4063 participants (including 2334 women) out of 6000 permanent residents who aged between 35 and 70 years [14]. Sampling was conducted based on the health center statistics and geographical divisions of urban and rural areas. Data collection principles were taught to recruited cohort staff. Interview and evaluations were performed by professional researchers. The cohort staff professions comprised of 14 researchers, including interviewer (n = 6), nutritionist (n = 2), physician (n = 1), nurse (n = 1), administration support officer (n = 1), field manager (n = 1), epidemiologist (n = 1), and biochemist (n = 1). The primary investigator supervised the study, which was conducted by the executive team and an academic panel [15]. Based on Fig. 1, Of the 2,334 women who consented to participate in BKNCD, 894 were menopaused (lack of menstruation for at least 12 months). Premature menopause was defined as menopause onset before the age of 40, while early menopause was defined as the onset of menopause between 40 and 44 years old. Normal menopause was defined as menopause after the age of 45 [16]. In this study, infertility was defined as failure to achieve pregnancy after one year of regular sexual intercourse without using contraceptives. Participants with factors and medical conditions that could affect age of menopause or hinder the menopausal definitions, including severe obesity (body mass index [BMI] greater than 35 kg/m^2^), bilateral oophorectomy, surgical menopause (hysterectomy), polycystic ovary syndrome, pregnancy and use of estrogen containing medications were excluded from the study.

### Data Preparation

Data collection was performed using a standard self-reported questionnaire and face to face interview. The survey questionnaire consisted of general information, history of chronic diseases, occupational history, BMI, socioeconomic status, drinking and smoking, history of fertility, information about the age at menarche, gravida, number of abortions, breastfeeding duration (month), and age of the first marriage (year). The socioeconomic status was assessed by obtaining information about participants’ travels (domestic or international trips), reading books, access to computer and internet, owning a car / motorcycle, and possessing household appliances (washing machine, dishwasher, vacuum cleaner and freezer). The socioeconomic status was evaluated by scoring the mentioned variables based on a 5-point Likert scale (level 1: poorest, level 2: Poor, level 3: moderate, level 4: good and level 5: richest). Anthropometric indices and blood pressure were measured for all participants. Anthropometric indices, including height, weight, waist and hip circumferences were measured by trained nurses based on the BKNCD cohort protocol [13]. All study procedures were approved by the Ethics Committee of the Hormozgan University of Medical Sciences (IR.HUMS.REC.1399.494). A written informed consent was obtained from all participants before inclusion in the evaluation.

### Data analysis

All data were analyzed using IBM SPSS-23.0 (IBM Corp., Armonk, NY, USA) software and a P < 0.05 was considered as statistically significant. Continuous variables with skewness and kurtosis between − 2 and + 2 were considered normally distributed [17]. Therefore, the normality of dependent variable (menopause age) was confirmed by skewness = 0.48 (standard error = 0.21) and kurtosis = 0.15 (standard error = 0.41). Variables were described using mean ± standard deviation (continuous) or number and percentage (categorical). Independent sample t-test was used to compare the means of the two independent groups and Chi-square test was used to determine whether there was a relationship between categorical variables. ANOVA was used to compare the means of continuous variables between more than two groups. Pearson correlation coefficient was used to determine the strength and direction of linear association between quantitative continuous variables.

Multivariable linear regression model was used to identify factors potentially associated with mean age at menopause. For this purpose, variables with P value less than 0.2 based on the univariable linear regression model (age at the first marriage, education, gravida, number of abortions, breast feeding duration, infertility, cardiovascular disease, thyroid disease, residence type, and socioeconomic status) were entered in the multivariable linear regression model.

Also, multinomial logistic regression model was used to determine the association between independent variables and the categorical dependent variable (premature menopause, early menopause and normal menopause). Then, variables with P value less than 0.2 based on the univariable multinomial logistic regression model (menarche age, gravida, number of abortions, age at the first marriage, stillbirth, tubal ligation, infertility, and socioeconomic status) were entered in the multivariable logistic regression model with menopause age category (premature menopause, early menopause and normal menopause) as dependent variable. Normal menopause category was set as reference category in this model.

## Results

**Fig. 2:**
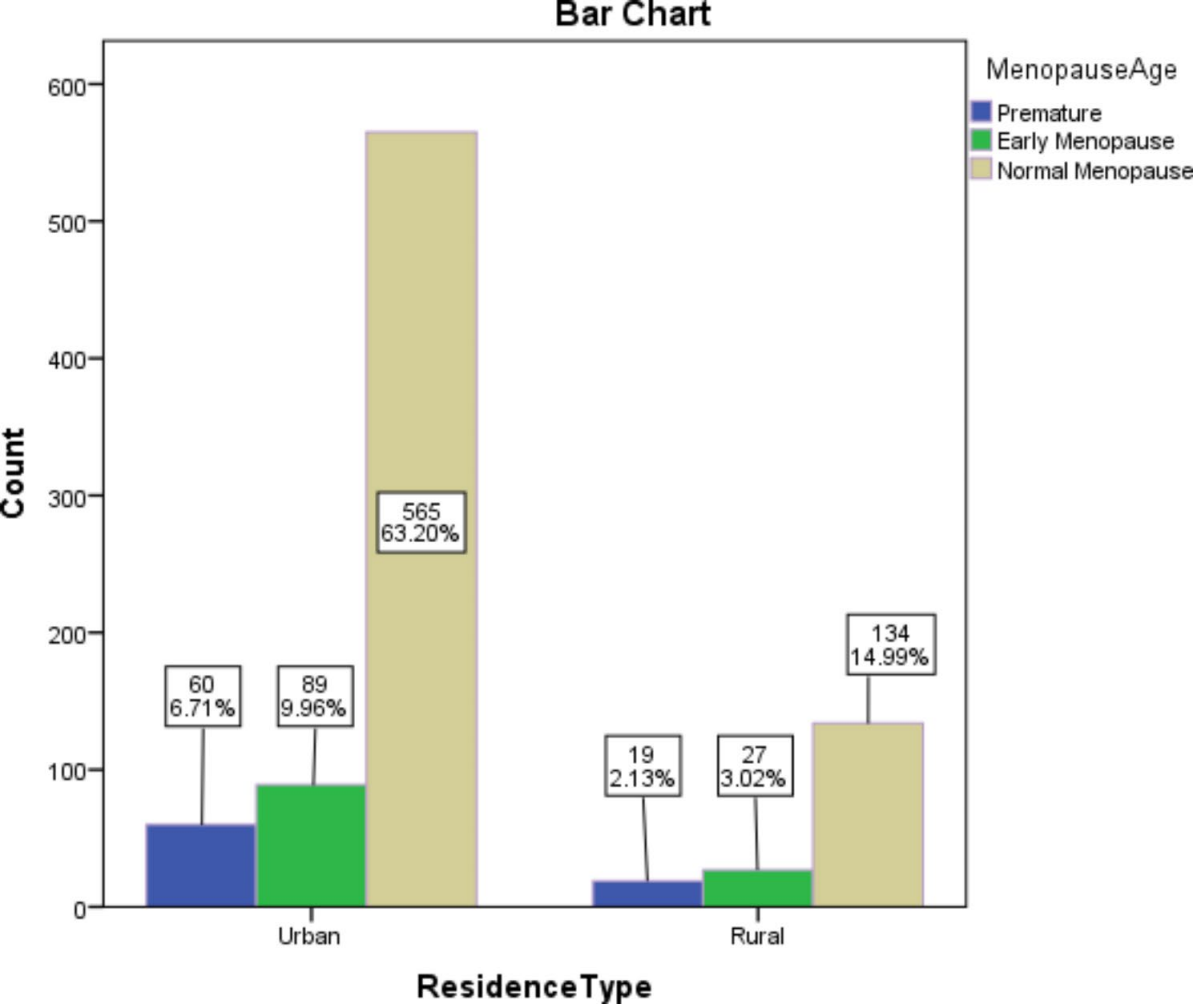
Frequency of menopause age by residence type

The study included 894 menopausal women within the age range of 35–70 years old. The mean age of natural menopause was 48.31 ± 6.34 years old. Table 1 summarizes the baseline characteristics of the study population. Figure 2 shows the frequency of premature menopause, early menopause, and normal menopause by residence type.

**Table 1:** Characteristics of the study population (n = 894)

Characteristics		N = 894
Age		57.32 ± 6.37
Having a history of stillbirth		170(19.8%)
Tubal ligation		265(29.6%)
Infertility		45(5.1%)
Diabetes		219(24.5%)
Hypertension		301(33.7%)
Cardiovascular disease		147(16.4%)
Thyroid disease		126(14.1%)
Depression		37(4.1%)
Smoking		134(15.0%)
Anthropometric index	Body Mass Index	27.89 ± 5.05
	Weight (Kg)	68 ± 13.5
	Height Cm	156 ± 6.37
Married status	Single(never married)	22(2.5%)
	Married	723(80.9%)
	Divorced/widow	149(16.9%)
Social economic status	Poor	357(39.9%)
	Moderate	180(20.1%)
	Rich	353(39.7%)
Residence Type	Urban	714(79.9%)
	Rural	180(20.1%)
Educational status	< 6(y/o)	827(92.5%)
	6–12(y/o)	54(6%)
	> 12(y/o)	13(1.55%)
Job status	housekeeper	822(91.9%)
	Employee	9(1%)
	other jobs	63(7%)
Body Mass Index	< 25	252(28.2%)
	≥ 25	642(71.8%)

Quantitative variables were described using mean ± standard deviation and categorical variables by number and percentage

The mean age of menopause based on premature, early and late menopausal stage among marital status, socioeconomic status, job, thyroid disease, diabetes, hypertension, cardiac disease, cardiovascular disease history, body mass index, smoking, still birth, infertility and tubal ligation categories are shown in Table 2. Based on the analysis presented in Table 2, there was no significant difference in overall menopause age between the evaluated variables (p > 0.05). Among the premature menopause group, the mean age of menopause was significantly higher among diabetes patients compared to non-diabetic (p = 0.043), while the mean age of menopause was significantly lower among women with the history of infertility compared to those without history of infertility (p < 0.001). However, among women with normal menopause age, age of menopause was significantly lower among women with the history of tubal ligation compared to women without the history pf tubal ligation (p = 0.030).

**Table 2:** Mean menopausal age based on categorical variables

Variable		Total (n = 894)		Premature menopause age (n = 79)		Early menopause age (n = 116)		Normal menopause age (n = 699)	
		Mean ± SD	P	Mean ± SD	P	Mean ± SD	P	Mean ± SD	P
Education (years)	< 6(y/o)	48.41 ± 6.36	0.251	34.25 ± 3.64	0.979	41.96 ± 1.55	0.592	51.01 ± 3.77	0.270
	6–12(y/o)	47.19 ± 6.15		34.29 ± 2.69		42.17 ± 1.47		50.12 ± 2.65	
	> 12(y/o)	46.69 ± 5.49		35.00 ± 0.00		42.75 ± 1.89		50.13 ± 2.90	
Gravida (number)^*^	< 7	47.41 ± 6.32	< 0.001	33.79 ± 4.04	0.209	42.17 ± 1.47	0.186	50.36 ± 3.39	< 0.001
	≥ 7	49.19 ± 6.20		34.82 ± 2.81		41.78 ± 1.65		51.46 ± 3.88	
Marital status	Single	48.50 ± 5.66	0.722	34.10 ± 3.20	0.639	41.71 ± 1.45	0.701	51.67 ± 3.66	0.423
	Married	48.39 ± 6.21		34.16 ± 3.19		42.07 ± 1.55		50.87 ± 3.64	
	Widow/Divorce	47.93 ± 7.08		34.61 ± 4.58		41.81 ± 1.66		51.28 ± 4.06	
Socioeconomic status	Poor	48.89 ± 6.46	0.060	34.23 ± 4.06	0.987	42.07 ± 1.57	0.530	51.40 ± 3.80	0.051
	Moderate	48.19 ± 6.25		34.40 ± 2.75		41.70 ± 1.59		50.96 ± 3.60	
	Rich	47.76 ± 6.24		34.24 ± 3.42		42.10 ± 1.53		50.46 ± 3.59	
Job	No	48.37 ± 6.41	0.516	34.20 ± 3.62	0.680	41.92 ± 1.55	0.167	51.03 ± 3.72	0.132
	Yes	47.96 ± 5.92		34.70 ± 2.87		42.50 ± 1.55		50.40 ± 3.59	
Thyroid disease	No	48.43 ± 6.33	0.161	34.22 ± 3.37	0.808	41.95 ± 1.53	0.389	51.01 ± 3.78	0.243
	Yes	47.58 ± 6.39		34.47 ± 4.26		42.31 ± 1.70		50.54 ± 3.20	
Diabetes	No	48.27 ± 6.43	0.684	33.82 ± 3.06	0.043	41.95 ± 1.57	0.588	50.93 ± 3.69	0.803
	Yes	48.47 ± 6.08		35.68 ± 2.93		42.13 ± 1.53		51.01 ± 3.79	
Cardiac Disease	No	48.30 ± 6.34	0.830	34.37 ± 3.56	0.461	41.99 ± 1.55	0.880	50.93 ± 3.66	0.727
	Yes	48.42 ± 6.36		33.44 ± 3.28		42.05 ± 1.62		51.07 ± 3.98	
CVD history	No	48.28 ± 6.37	0.724	34.37 ± 3.56	0.461	41.97 ± 1.55	0.660	50.94 ± 3.67	0.853
	Yes	48.48 ± 6.20		33.44 ± 3.28		42.13 ± 1.60		51.01 ± 3.91	
Body mass index (kg/m2)	< 25	48.47 ± 6.84	0.707	34.41 ± 3.89	0.641	42.35 ± 1.45	0.186	51.41 ± 4.01	0.110
	25–30	48.40 ± 6.02		34.61 ± 2.51		42.02 ± 1.58		50.86 ± 3.66	
	> 30	48.05 ± 6.31		33.71 ± 4.14		41.65 ± 1.57		50.67 ± 3.46	
Smoking	No	48.28 ± 6.31	0.696	33.97 ± 3.57	0.108	42.04 ± 1.55	0.479	50.91 ± 3.62	0.469
	Yes	48.51 ± 6.58		35.61 ± 3.05		41.73 ± 1.62		51.19 ± 4.21	
Stillbirth	No	48.49 ± 5.90	0.801	34.98 ± 2.81	0.298	42.05 ± 1.58	0.480	50.85 ± 3.65	0.067
	Yes	48.36 ± 7.21		34.23 ± 2.79		41.75 ± 1.61		51.52 ± 4.06	
Infertility	No	48.45 ± 6.11	0.051	34.84 ± 2.83	< 0.001	42.00 ± 1.58	1.000	50.94 ± 3.71	0.306
	Yes	46.56 ± 9.51		29.13 ± 5.22		42.00 ± 1.23		51.61 ± 3.72	
Tubal ligation	No	48.31 ± 6.60	0.948	33.82 ± 3.62	0.073	42.02 ± 1.58	0.782	51.15 ± 3.82	0.030
	Yes	48.34 ± 5.70		35.41 ± 3.07		41.93 ± 1.51		50.49 ± 3.40	

*The mean of gravida was 6.8 and its median was 7. Independent t-test was used to compare mean age at menopause between two groups; The ANOVA test was used to compare the mean age at menopause in more than two groups. SD: Standard deviation, CVD: Cardiovascular disease

Findings of the multivariable linear regression model (Table 3) indicated that gravida, cardiovascular disease, type of residency, and socioeconomic status were the most important variables that affected age at menopause. These findings indicated that for each unit increase in gravida, the mean menopause age increased by 0.29 years (B = 0.29; P = 0.010) after adjusting for other variables. The mean menopause age among women with a history of cardiovascular disease was 1.89 years lower compared to their healthy counterparts (B= -1.89; P = 0.035). The mean menopause age among women who lived in urban area was 1.38 years lower compared to women who lived in rural area (B= -1.38; P = 0.013). The mean menopause age among poor women was 1.47 years lower compared to rich women (B= -1.47; P = 0.010).

**Table 3:** Linear regression model to identify factors potentially associated with age at menopause

Variable	Univariable Models		P value	Multivariable Model		P value
	B	95% CI		B	95% CI	
First marriage age (Years old)	-0.07	-0.16,0.02	0.112	0.03	-0.09,0.10	0.942
Education (years)	0.17	0.04,0.29	0.009	0.06	0.08,0.21	0.381
Gradiva	0.29	0.16,0.43	< 0.001	0.29	0.04,0.41	0.010
Number of abortions	0.39	-0.02,0.80	0.061	0.08	-0.40,0.57	0.724
Breast feeding Duration	0.007	0.00,0.01	0.024	0.002	0.00,0.01	0.714
BMI (< 25) (Ref: ≥25)	0.22	-0.71,1.15	0.641	-	-	-
Having a history of stillbirth (Ref: No history)	-0.13	-1.17,0.91	0.801	-	-	-
Tubal ligation (Ref: No)	0.03	-0.88,0.94	0.948	-	-	-
Infertility (Ref: No)	-1.89	-3.79,0.01	0.051	-0.25	-2.51,2.00	0.827
Diabetes (Ref: No)	-0.20	-1.77,0.17	0.684	-	-	-
Hypertension (Ref: No)	-0.37	-1.51,0.25	0.407	-	-	-
Cardiovascular disease(Ref: No)	-1.20	-1.32,0.02	0.064	-1.89	-2.98,-0.09	0.035
Thyroid disease (Ref: No)	-0.86	-2.05,0.34	0.161	-0.29	-1.51,0.92	0.640
Depression (Ref: No)	-0.66	-2.76,1.42	0.532	-	-	-
Smoking(Non-smokers) (Ref: Smokers)	-0.23	-1.39,0.93	0.696	-	-	-
Marital status (Not married) (Ref: Married)	-0.38	-1.44,0.67	0.480	-	-	-
Residence type (urban) (Ref: Rural)	-0.77	-1.81,0.27	0.146	-1.38	-2.46,-0.29	0.013
Socioeconomic status(poor) (Ref: Rich)	-0.95	-1.79,-0.10	0.028	-1.47	-2.81,-0.07	0.010
Socioeconomic status (moderate) (Ref: Rich)	-0.15	-1.19,0.88	0.767	-0.31	-1.48,-0.84	0.593

Dependent Variable: Menopause Age; Overall sample size = 894; Ref: Reference category; CVD: Cardiovascular disease, BMI: Body mass index; Variables that had a P value ≤ 0.2 in the univariable models, were added in multivariable model

Based on the analysis presented in Table 4, the odds of premature menopause were 0.53 less for women who did not have a history of stillbirth compared to their counterparts who had the history of stillbirth after adjusting for other variables (Odds ratio = 0.469; P = 0.010). Moreover, for one unit increase in gravida, the odds of premature menopause reduced by 0.10 after adjusting for other variables (Odds ratio = 0.899; P = 0.048).

**Table 4:** Multivariable multinomial logistic regression model to identify factors potentially associated with menopause

Variable		Premature menopause age (n = 79)			Early menopause age(n = 116)		
		Odds ratio	95% CI	P value	Odds ratio	95% CI	P value
Menarche age		1.00	0.85–1.18	0.969	0.89	0.78–1.03	0.112
Gravida		0.90	0.81-1.00	0.048	0.98	0.90–1.08	0.722
Number of abortions		1.07	0.81–1.41	0.631	0.88	0.68–1.14	0.320
Age at the first marriage		0.99	0.93–1.04	0.615	1.01	0.97–1.06	0.648
Stillbirth	No	0.47	0.26–0.83	0.01	0.805	0.45–1.46	0.474
	Yes	Reference			Reference		
Tubectomy	No	1.01	0.59–1.73	0.966	1.30	0.81–2.09	0.276
	Yes	Reference			Reference		
Infertility	No	0.71	0.23–2.19	0.554	0.866	0.29–2.60	0.797
	Yes	Reference			Reference		
Socioeconomic status	Poor	0.90	0.52–1.55	0.700	0.89	0.55–1.45	0.649
	Moderate	0.89	0.44–1.78	0.738	0.78	0.45–1.36	0.384
	Rich	Reference			Reference		

women with normal menopause were considered the reference category; a P value < 0.05 was considered as statistically significant

## Discussion

In this study, the risk factors for age at menopause were investigated in general and by classifying early, premature and natural menopause. This study adds evidence to the ongoing discussions about factors affecting age at menopause based on the data obtained from Bandar-e-Kong cohort in south of Iran. In our study, the mean age of menopause was 48.31 ± 6.34 years in total population. Overall, the reported mean age of menopause in our study was similar to the reported age of menopause in other studies in Iran [8, 11, 18–22]. The age at menopause in our study was lower than the menopause age reported in western countries [23–25] and China (50.53 ± 6.57), but was higher than the reported menopause age in Punjab, India (47.9 ± 3.2) [26, 27]. This difference might be attributed to the variations in genetic and environmental factors [28]. In this study we found no association between marital status and any of the menopause categories, which was in line with the findings of some previous studies [19, 29, 30]. Studies that found a significant role for marital status in age at menopause related this effect to a better socioeconomical status, better family support, higher hygiene level, and better quality of life in married women compared to single women [30, 31]. Furthermore, as pregnancy and child birth were found to be related to older age at menopause, married women have more predisposing factors for older age at menopause [30]. The results of our study also showed a significant relationship between age at menopause and gravida. The findings of previous studies on the relationship between gravida and age at menopause were also controversial [32, 33]. Shin et al. (2017) reported that delivery was related to menopause age, which confirmed the result of our study [34]. The possible mechanism for the effect of pregnancy on menopausal age is the induced anovulation during pregnancy [35]. Similar effect was observed in terms of oral contraceptive use [36]. On the other hand, other confounders might have a stronger effect on menopause age. Based on the mentioned reasons, marital status might not be considered as a direct predictor of age at menopause but can be considered as a combination of preventive factors that affect age at menopause. In our study, socioeconomic and gravida were significantly related to overall age at menopause. Therefore, it can be suggested that in our study other factors including family support or quality of life were not different between married and single/divorced women; therefore, these factors resulted in a non-significant relationship between marital status and age at menopause.

The results of our study showed no significant relationship between BMI and menopausal age. The results of previous studies in terms of the relationship between BMI and menopausal age were controversial. While some studies reported no relationship between BMI and age at menopause [26], others reported increased risk for early menopausal among underweight women and increased risk for delayed menopause among overweight and obese women [31, 37]. A reason for the difference between the findings of our study and previous studies might be related to the higher prevalence of overweight and obesity compared to underweight among our study population. Unequal number of women with different BMI categories might be the cause for non-significant difference in age at menopause between the groups as the sample size was not determined based on BMI subgroups.

Our study showed that smoking was not related to age at menopause. Previous studies have indicated that cigarette smoking can lead to premature menopause [38, 39]. The exact mechanism for the effect of smoking on menopause is not yet clear. It is hypothesized that the toxins inhaled during smoking might affect FSH and E2 levels [40]. However, not all studies have proven the effect of smoking on menopausal age [41]. The reason for this difference might be attributed to the extent and duration of smoking as well as the confounding effect of passive smoking [40, 42]. Furthermore, our study included a small number of smokers, which might be the reason for failure to determine an association between smoking and menopausal age.

Our study also showed that the mean menopause age among women with a history of cardiac disease was 2.131 years lower compared to their healthy counterparts, and the mean menopause age among women with a history of CVD was 2.651 years lower compared to their healthy counterparts. As this study was cross-sectional, the observed association might not indicate causation. Previous studies have reported that premature menopause was associated with increased risk for cardiovascular diseases [43–45]. Therefore, it can be hypothesized that CVD might not be a predictor of menopause age but may be considered as a consequence of menopause. As women with the history of early and premature menopause might experience CVD at an earlier age compared to those with natural menopause, more CVD incidents were observed among women with early and premature menopause.

Our study also showed that mean menopause age among women who lived in urban area was 1.339 years lower compared to women who lived in rural area (B= -1.339; P = 0.019). Furthermore, the mean menopause age among poor women was 1.14 years lower compared to rich women (B= -1.140; P = 0.019). Previous studies have reported inconsistent findings in relation to the age of premature menopause among women living in rural and urban areas [46–48]. A reason for the difference in the findings of studies in relation to sociodemographic variables and premature menopause might be related to the difference in the accessibility to health care services, different concerns and attitudes about menopause, and different sample sizes in the studies.

The mean age of natural menopause in Bandar-e-Kong cohort population was lower than Hamedan province (49.6 ± 4.0 years) [49]. The findings of our study in terms of the association between infertility and premature menopause was in line with the findings of a previous study [50]. On the other hand, age at menopause was significantly lower in infertile women compared to those with normal fertility only among the premature menopause group. This finding might indicate that as infertility is a condition with heterogeneous etiology, some etiologies of infertility might affect age at menopause. Therefore, it is recommended that further studies focus on the relationship between infertility etiologies and age at menopause.

One of the limitations of our study was that data distribution was not balanced for some variables. hence, the power of the statistical models may be affected by imbalanced in the dataset. Also, this study identified predictors of age at menopause based on a cross-sectional study. Therefore, these relationships may not indicate causation. Prospective studies are needed to evaluate the effects of exposure to these predictors during premenopausal period on age at menopause. Another limitation of our study was collecting data based on self-reports, which can be subject to recall bias. Moreover, remembering the exact time of menopause might be subject to recall bias as well. This study suggests that duration of breastfeeding, smoking, menarche age, underlying diseases, and BMI did not affect the age of menopause. Prospective studies are needed to evaluation the effects of these factors on menopausal age.

### Electronic supplementary material

Below is the link to the electronic supplementary material.

Supplementary Material 1

## Data Availability

The datasets generated and/or analyzed during the current study are not publicly available due to university rules and regulation of data ownership but may be accessible through official written request for the corresponding author.
