# Correction to: Factors predicting age at menopause among Iranian women in the Bandare-Kong cohort study (a cross-sectional survey of PERSIAN cohort study)

**DOI:** 10.1186/s40695-023-00091-4

**Published:** 2023-11-20

**Authors:** Maryam Azizi Kutenaee, Sareh Dashti, Shideh Rafati, Mehrsa Moannaei, Mojtaba Masoudi, Abdolazim Nejatizadeh, Mehdi Shahmoradi, Nasibeh Roozbeh

**Affiliations:** 1 Fertility and Infertility Research Center, Hormozgan University of Medical Sciences, Bandar Abbas, Iran; 2 Department of Public Health, Faculty of Paramedicine, Mashhad Medical Sciences, Islamic Azad University, Mashhad, Iran; 3 Department of Midwifery, Faculty of Nursing and Midwifery, Mashhad Medical Sciences, Islamic Azad University, Mashhad, Iran; 4 Social Determinants in Health Promotion Research Center, Hormozgan University of Medical Sciences, Bandar Abbas, Iran; 5 Department of Gynecology and Obstetrics, School of Medicine, Fertility and Infertility Research Center, Hormozgan University of Medical Sciences, Bandar Abbas, Iran; 6 Fatemiyeh Shiraz Institute of Higher Education, Shiraz, Iran; 7 Molecular Medicine Research Center, Hormozgan Health Institute, Hormozgan University of Medical Sciences, Bandar Abbas, Iran; 8 Endocrinology and Metabolism Research Center, Hormozgan University of Medical Sciences, Bandar Abbas, Iran; 9 Mother and Child Welfare Research Center, Hormozgan University of Medical Sciences, Bandar Abbas, Iran

Following publication of the original article [1], we have been notified that the author’s name was published incorrectly. It was - Mehrsa moannaie. It should be - Mehrsa Moannaei.

The original article was updated.
