# Supplementary material for: Factors predicting age at menopause among Iranian women in the Bandare-Kong cohort study (a cross-sectional survey of PERSIAN cohort study)
Source: Womens Midlife Health. 2023 Aug 29;9:5. doi: 10.1186/s40695-023-00088-z (PMC10463461; doi:10.1186/s40695-023-00088-z)
Supplement: Supplementary file 1 — Supplementary Material 1 [file 40695_2023_88_MOESM1_ESM.docx]

Response to reviewer comments

Reviewer reports:

In this revision you did not include the grammatical changes requested in the copy-edited version of the manuscript. The copy edited version of the prior submission is attached. Please review this document and make the changes as requested in the current version and resubmit the manuscript.

**Response:** We thank the reviewer for the comment. The edited version of article with track change and without track change is attached

In addition, please make these additional edits in the revised sections of the current manuscript.

1) Abstract Results
Change " Among the premature menopause group, the mean age at menopause was
significantly higher among diabetic women in premature menopause age group compared to non-diabetic
group...."  to " Among the premature menopause group, the mean age at menopause was
significantly higher among women with diabetes compared to women without diabeties ..."
group (35.68±2.92 vs. 33.82±3.06; P = 0.043)

**Response:** Was revised and highlighted in main text.

2) Background, second to last sentence
Change "According to the importance of early menopause and premature menopause, it is necessary that the related predictive factors for these conditions be separately evaluated. "
to "Given the importance of early menopause and premature menopause, separate evaluation of factors elated to  these conditions is necessary. "

**Response:** Was revised and highlighted in main text.

3) Results second paragraph first line "menopausal state" should be "menopausal stage"

**Response:** Was revised and highlighted in main text

4) Table 2 should include education and gravida -- important variables in Table 3. Also please place variables in the same order in all tables.

**Response:**

In Table 3, education and gravida were quantitatively entered into the regression model. While in Table 2, the average age of menopause has been calculated by different subgroups of each categorical variable. However, according to your valuable opinion, we added these two variables in a categorical form in Table 2.

5)Discussion first paragraph
a) Change "In this study we did not find any association between marital status and neither of the menopause categories, which was in line with the findings of some previous studies "
to 'In this study we did not find an association between marital status and any of the menopause categories, in line with the findings of some previous studies ..."

b) Change "Similar effect was observed in terms of oral
contraceptive use (36). On the other hand, other confounders might have a stronger effect on menopause age. Based on the mentioned reasons, marital status might not be considered as a direst predictor of age at menopause but can be considered as a combination of preventive factors that affect age at menopause.
In our study, socioeconomic and gravida were significantly ..."
To "A similar effect was observed in terms of oral contraceptive use (36). As these other confounders might have a stronger effect on menopause age than marital status, marital status might not be  a direct predictor of age at menopause but may reflect a combination of preventive factors that affect age at menopause.
In our study, socioeconomic status and gravida were significantly ..."

**Response:** A and b comments were revised and highlighted in main text.

6) Discussion paragraph 3 Change "Our study showed that smoking"  to "Our study found that smoking"
and CHange "However, not all studies have proven the effect of smoking ..." to "However, not all studies have found an effect of smoking ..."

**Response:** Was revised and highlighted in main text

7) Discussion paragraph 4
CHANGE " Therefore, it can be hypothesized that the CVD might not be a predictor of menopause age but be
considered as a consequence of menopause and as women with the history of early and premature
menopause might experience CVD at an earlier age compared to those with natural menopause, more
CVD incidents were observed among the women with early and premature menopause."
TO " Therefore, it can be hypothesized that CVD might be  a consequence of menopause and as women with  a history of early and premature
menopause might experience CVD at an earlier age compared to those with a normative age at menopause, more
CVD incidents were observed among the women with early and premature menopause."

**Response:** Was revised and highlighted in main text
